# The effect of maternal antibiotic use in sows on intestinal development in offspring

**DOI:** 10.1093/jas/skaa181

**Published:** 2020-06-01

**Authors:** Astrid de Greeff, Dirkjan Schokker, Petra Roubos-van den Hil, Peter Ramaekers, Stephanie A Vastenhouw, Frank Harders, Alex Bossers, Mari A Smits, Johanna M J Rebel

**Affiliations:** 1 Wageningen Bioveterinary Research, Lelystad, The Netherlands; 2 Wageningen Livestock Research, Wageningen, The Netherlands; 3 Trouw Nutrition R&D, Amersfoort, The Netherlands

**Keywords:** antibiotic, gut development, immune response, microbiota, piglets, transgenerational

## Abstract

The objective of this study is to investigate the effect of a maternal antibiotic administration during the last week of gestation on the early life intestinal development in neonatal piglets. Colonization of the gut with bacteria starts during birth and plays a major role in the intestinal and immunological development of the intestine. We demonstrate that maternal interventions induced changes in the sows (*n* = 6 to 8 per treatment) fecal microbiota diversity around birth (*P* < 0.001, day 1). Whole-genome microarray analysis in small intestinal samples of 1-d old piglets (*n* = 6 to 8 per treatment) showed significantly expressed genes (*P*_adj_ < 0.05) which were involved in processes of tight junction formation and immunoglobulin production. Furthermore, when performing morphometry analysis, the number of goblet cells in jejunum was significantly (*P* < 0.001) lower in piglets from amoxicillin administered sows compared with the respective control piglets. Both significantly expressed genes (*P*_adj_ < 0.05) and significant morphometry data (jejunum *P* < 0.05 and ileum *P* < 0.01) indicate that the crypts of piglets from amoxicillin administered sows deepen around weaning (day 26) as an effect of the amoxicillin administration in sows. The latter might imply that the intestinal development of piglets was delayed by maternal antibiotic administration. Taken together, these results show that maternally oral antibiotic administration changes in early life can affect intestinal development of the offspring piglets for a period of at least 5 wk after the maternal antibiotic administration was finished. These results show that modulation of the neonatal intestine is possible by maternal interventions.

## Introduction

Maturation of the gastrointestinal tract of piglets is a multifactorial process that is important for the health of piglets in production systems (Penders et al., 2006; Mulder et al., 2009; Langley-Evans, 2015; Nobel et al., 2015). Here, we aimed to improve health of piglets via maternal interventions. Studies in humans and animals indicate that maternal diet in gestation and lactation influences intestinal development and health in offspring. Intestinal microbiota contributes significantly to immunological development and metabolic health (Cox et al., 2014). Shifts in the composition of the microbiome, especially at young age, can lead to perturbation of immune regulatory networks in the intestine that affect health (Schokker et al., 2014; Benis et al., 2015; Merrifield et al., 2015). This implies that changes and interventions in the early life development of the gut could have long-term effects on the competence of the host immune system.

It was shown previously that a single antibiotic treatment in piglets directly after birth led to significant changes in microbiome composition of piglets that persisted over time (Janczyk et al., 2007; Schokker et al., 2014, 2015). Antibiotic-treated pigs developed into a different homeostatic state than control piglets, indicating that early life interventions can have long-lasting effects (Schokker et al., 2015). Our hypothesis is that maternal interventions will influence the intestinal immune development of the offspring. Therefore, the objective of this study is to investigate the effect of a maternal antibiotic (amoxycillin) treatment during the last week of gestation on the early life intestinal development in neonatal piglets.

## Materials and Methods

### Ethical statement

The established principles of laboratory animal use, the European Union, and Dutch laws related to animal experiments were adhered to in this study. The Dutch Commission for Animal Studies approved the study under 2012.III.05.041. The animal experiment was approved by the Ethical Committee of Utrecht University (the Netherlands), in accordance with the Dutch law on animal experiments. Animal suffering was not expected within this particular experiment due to the nature of the intervention (amoxicillin treatment).

### Experimental design, housing, and diet

Thirty-one Hypor gilts and sows (and their offspring) divided over three different batches were allotted to one of two treatments based on BW, backfat thickness and parity 4 wk before farrowing (day 87 of gestation) (de Greeff et al., 2015), all these animals were included for the zootechnical parameters. Only a limited number of sows were included for molecular analyses (*n* = 6 to 8 per group). Both treatment groups received standard gestation and lactation feed (Supplementary Table S1, values were obtained by chemical analysis). The sows received a daily dosage of Paracillin SP (amoxicillin) (MSD Animal Health, Boxmeer, The Netherlands) added to the diet (Supplementary Table S1), from 1 wk before expected day of farrowing until farrowing, at an inclusion rate of 15 mg/kg BW. From farrowing till weaning, sows were fed standard lactation diet without antibiotics. Sows were housed at Trouw Nutrition Swine Research Centre in gestation group housing and lactation departments. During lactation, sows were housed individually. Sows had free access to water and were fed according to a standard feeding scheme. Feed intake was recorded using automated feeders. Farrowing was induced in sows that did not farrow naturally on day 114 of gestation. The sows were weighed at the start of the trial (day 87 of gestation), on arrival to the lactation room (day 108 gestation), on days 2 and 7 after farrowing and at weaning (day 26). Colostrum sample was collected after the first piglet was born and milk was collected 7 d after farrowing. To measure the treatment effect on microbiota, fecal samples from sow were collected on 7 d before farrowing, day of farrowing and 7 d after farrowing from three sows of the both batches (resulting, six to eight sows per treatment). Vaginal swabs were collected after the first piglet was born for microbiota analysis. An overview of sow samples included in downstream analyses is shown in Table 1. The mortality of the piglets was recorded.

**Table 1.** Overview of the number of sampled sows and piglets used for downstream analyses^1^

Sow	Microbiota					
Time point (d)^2^	Amox^3^	Control				
−7	8	6				
0	6	6				
1	7	3				
7	8	6				
**Piglet**	**Microbiota**		**Gene expression**		**Histomorphometry**	
**Time point (d)4**	**Amox**	**Control**	**Amox**	**Control**	**Amox**	**Control**
1	7	4	4	6	5	4
7	7	7	4	6	8	6
W	6	6	4	3	7	6
W4	6	5	4	5	8	7
W28	6	4	4	3	7	4

^1^Includes only the samples that passed quality control.

^2^ −7, 7 d before starting the antibiotic treatment.

^3^Amox refers to the piglets born from sows that received amoxicillin.

^4^W refers to weaning.

Piglets were weighed immediately after birth, at 24 h (20 to 25 h), 7 d, at weaning (day 26) and 4 wk after weaning (day 54). On days 1 and 7, at weaning, 4 d after weaning (day 30) and 4 wk after weaning (day 54) one apparently healthy male piglet from each sow (*n* = 6 to 8) was selected, based on the average BW of the piglets and in good health and euthanized by intravenous administration of Euthasol (24 mg/kg BW) and subsequent exsanguination (total 30 piglets) to collect intestinal digesta and intestinal tissue samples. Intestinal digesta was collected from jejunum to analyze the microbiota. Intestinal scrapings were collected from ileum and jejunum for gene expression analysis. Sections of intestinal tissue from jejunum and ileum were spread on cork and fixed in formalin for histological morphometric analysis. An overview of piglet samples included in downstream analyses is shown in Table 1. Blood serum was collected to analyze systemic responses. To determine whether the milk from sow can potentially carry the amoxicillin traces provided to the sows, the amoxicillin levels in milk collected shortly after farrowing were determined by high-performance liquid chromatography (RIKILT, Wageningen, the Netherlands). Two out of 15 treated sows showed low (2.8 and 4 µg/kg) concentrations of amoxicillin in the milk, the other 13 samples were below detection levels, making a direct effect of amoxicillin traces via milk very unlikely.

### Analysis of microbiota composition and diversity

Microbiota diversity and composition was determined in sow feces, sow vaginal swabs, and piglet jejunal digesta samples. These collected samples were kept on dry-ice and further stored at −80 °C until analysis. To extract DNA, samples were mixed in a 1:1 ratio with cold PBS and centrifuged for 5 min at 4 °C at 300 × *g*, the centrifuging could potentially skew the microbiota distribution by excluding bacteria that adhere to particulate material. Supernatant was collected and centrifuged for 10 min at 4 °C at 9,000 × *g* (Eppendorf). Mechanical shearing was carried out on the pellets containing the bacterial load in Lysing Matrix B tubes using the FastPrep-24 (MP Biomedicals, Solon, OH) following manufactures protocol, i.e., three times 30 s at a speed of 30 Hz. Thereafter, DNA was extracted using the “QIAamp DNA stool minikit” (Qiagen, Valencia, CA) according to manufacturers’ instructions. Both, quality and quantity of DNA were checked using the NANOdrop (ND1000, Agilent Technologies, Santa Clara, CA). For bacterial amplicon library preparation, PCR was performed to amplify (20 cycles) the 16S rRNA gene hypervariable region V3 fragment using forward primer V3_F (CCTACGGGAGGCAGCAG) and reverse primer V3_R (ATTACCGCGGCTGCTGG) (Schokker et al., 2018a). The amplicons were checked on agarose gel. Thereafter, the amplicons from each samples were bar-coded with Illumina adapters and sequenced using paired-end sequencing, 2 × 150 bp technology on a MiSeq sequencer (Illumina, San Diego, CA) at a sequencing depth in the range of 196K to 1.2M read-pairs per sample (median 670,647 read-pairs per sample). One sample that did not pass the quality control was excluded. The adapter and primer-clipped, filtered, and error-corrected sequence data are available as a phyloseq object at https://doi.org/10.5281/zenodo.3602861, which can be directly loaded into R by using the ‘phyloseq’ package (McMurdie and Holmes, 2013).

The phyloseq object creation and statistical analyses were performed in R 3.5.1 (R Core Team, 2014). Briefly, the amplicon sequences were quality filtered, primer-trimmed, error-corrected, dereplicated, chimera-checked, and overlapping R1 and R2 sequences were merged using the dada2 package (v.1.4.0; Callahan et al., 2016). By using the standard parameters except for *TruncLength* = 140,100 and minOverlap = 10), and reads were classified with the SILVA v.132 classifier (Quast et al., 2013). The statistical analyses of the taxonomic distributions were performed with the *phyloseq* (version 1.28.0) (McMurdie and Holmes, 2013) and *vegan* (version 2.5 to 6) (Oksanen et al., 2019) packages. Prior to analyses, the data were rarefied to 17,192 per sample (*rarefy_even_depth,* rngseed=12345) to allow diversity comparisons and the final dataset contained 5,966 taxa.

To determine the (semi) quantitative level of microbial load per sample (1 mL), qPCR was performed. Briefly, qPCR was performed using 1:10,000 dilution of isolated DNA from the intestinal samples using the 16S rRNA gene-derived primers as described above and Powr Sybr Green PCR Master mix (Applied Biosystems) with the following PCR conditions: 10 min at 95 °C, 40× (15 s at 95 °C, 30 s at 59 °C, 36 s at 72 °C), followed by a dissociation curve using the ABI7500 (Applied Biosystems). To allow standardization of the amount of 16S rRNA and detect potential amplification inhibiting factors, an internal control is added to each reaction, to determine the PCR efficiency of each sample. This internal control, 1 ng of an internal control of nonrelated DNA (luciferase gene), was spiked to the samples and amplified in a separate qPCR using primers JR331 CCTGAAGTTCATCTGCACCA and JR328 CTTGTAGTTGCCGTCGTCCT with the following PCR conditions: 10 m at 95 °C, 40× (15 s at 95 °C, 30 s at 58 °C, 36 s at 72 °C), followed by a dissociation curve using the ABI7500 (Applied Biosystems). The (semi) absolute level of microbial load per sample was calculated by dividing the amount of 16S-amplicon signal by the amount of internal control (Green Fluorescent Protein). Data analysis was done using Graphpad software Prism (version 8.2.1) with a two-way ANOVA following *y* = time-effect × treatment-effect + residual errors with Bonferroni post hoc testing.

### Gene expression analysis of intestinal tissue

Total RNA was extracted from 50 to 100 mg jejunum tissue scrapings. Samples (representing an individual piglet) were homogenized using a TissuePrep Homogenizer in 5 ml TRIzol reagent (Life Technologies, Carslbad, CA). The homogenate was centrifuged for 5 min at 21,000 × *g*. Three hundred and fifty microliters of supernatant were used to isolate RNA using the Direct-zol kit (Zymo Research, Irvine, CA) according to instructions of the manufacturer. Quality control in terms of quality and quantity of the isolated RNA from each sample was performed on the BioAnalyser (Agilent Technologies) and Nanodrop (Agilent Technologies). Labeling (Cyanine-3), hybridization, scanning, and feature extraction of microarrays was performed as described before (Schokker et al., 2018b), porcine (one-color) microarrays were ordered at Agilent Technologies. Microarray data were submitted to NCBI GEO under accession number GSE115178. The data were analyzed by using R (v3.0.2) by executing different packages, including Linear Models for Microarray and RNA-Seq Data (**LIMMA**) and arrayQualityMetrics (Kauffmann et al., 2009). The data were read in and background corrected (method = “normexp” and offset = 1) with functions from the R package LIMMA (Ritchie et al., 2015) from Bioconductor (Gentleman et al., 2004). Quantile normalization of the data was done between arrays. The duplicate probes mapping to the same gene were averaged (‘avereps’) and subsequently the lower percentile of probes were removed in a three-step procedure: (1) get the highest of the dark spots to get a base value, (2) multiply by 1.1, and (3) the gene/probe had to be expressed in each of the samples in the experimental condition. The contrasts between piglets born from amoxicillin treated sows and control sows were studied for each time point. Genes were denoted as significant when the adjusted *P*-value, multiple testing correction method was False Discovery Rate, was below 0.05 and the log fold change was below −1.5 or above 1.5. The Database for Annotation, Visualization, and Integrated Discovery (DAVID, version 6.8) was used to perform Functional Annotation Clustering (**FAC**) for the different contrasts. The up- and downregulated genes, here adjusted *P*-value < 0.05 and log fold change below −1.0 or above 1.0, were separately analyzed. The fold change threshold was more lenient here, because we wanted to start with a higher number of (annotated) genes in order to have a sufficient number of genes to investigate the affected biological processes.

### Histomorphometric analysis of intestinal tissue

Intestinal tissues samples were collected in formalin. Formalin-fixed samples were embedded in paraffin, sectioned at a 5-μm thickness, and stained with hematoxylin and eosin for histologic examination. The villus length and crypt depth were determined. These morphometric measurements were performed by using the image analysis software Image Pro Plus 7 (Media Cybernetics, Silver Spring, MD). Each measurement of villus crypt ratio was determined in triplicate, on three representative locations per slide, the average was included for analysis.

To determine the proliferating cells in the tissue sections, proliferating cell nuclear antigen (**PCNA**) staining, a cell proliferation marker expressed by cells throughout the S to M phases of the cell cycle (Yu et al., 1992), was carried out. PCNA was detected as described before (de Greeff et al., 2016). Data analysis was performed by using the Graphpad software Prism (version 8.2.1) with a two-way ANOVA following *y* = time-effect × treatment-effect + residual errors with Bonferroni post hoc testing.

## Results

### Effect of antibiotic treatment on performance

**Figure 1.**
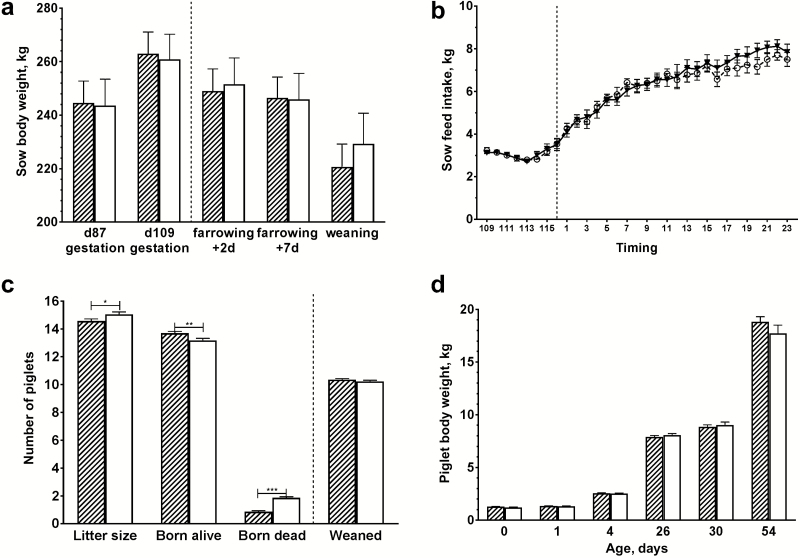
BW of sows and piglets, feed intake of sows and reproductive traits. BW of sows (panel a); feed intake of sows (panel b); reproduction traits of sows (panel c); BW of piglets are depicted (panel d). Open white bars represent the control group, hatched bars represent the amoxicillin-treated group. The hatched line indicates the moment of farrowing; error bars indicate standard error of the mean; * *P* < 0.05; ** *P* < 0.01; *** *P* < 0.001.

Antibiotic treatment did not significantly affect sow weight nor feed intake (Figure 1a and b). However, a coincidental difference in reproduction parameters was found between treatment groups, where sows in the antibiotic group had significantly smaller litter sizes, higher number of piglets born alive and a lower number of stillborn piglets (Figure 1c). Antibiotic treatment did not affect weight of piglets until 54 d of age when the experiment ended (Figure 1d).

### Effect of maternal antibiotic treatment on microbiota of sows and their offspring

**Figure 2.**
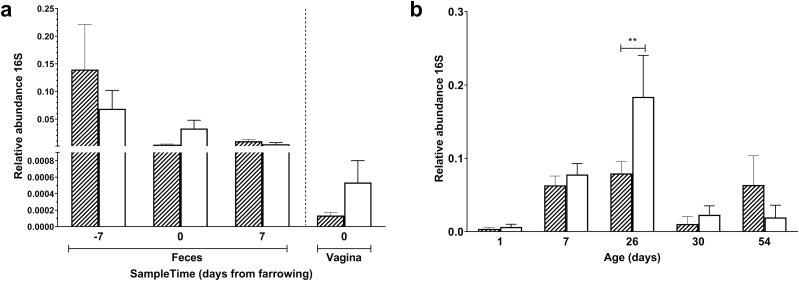
Abundance of microbiota as determined using a quantitative PCR on 16S rDNA genes. qPCR was performed on fecal and vaginal samples from sows (panel a) and jejunal content of piglets (panel b). Quantities were expressed relative to an internal PCR control (GFP) to correct for putative inhibition in individual samples. Open white bars represent the control group, hatched bars represent the amoxicillin-treated group; error bars indicate standard error of the mean; ** *P* < 0.01.

**Figure 3.**
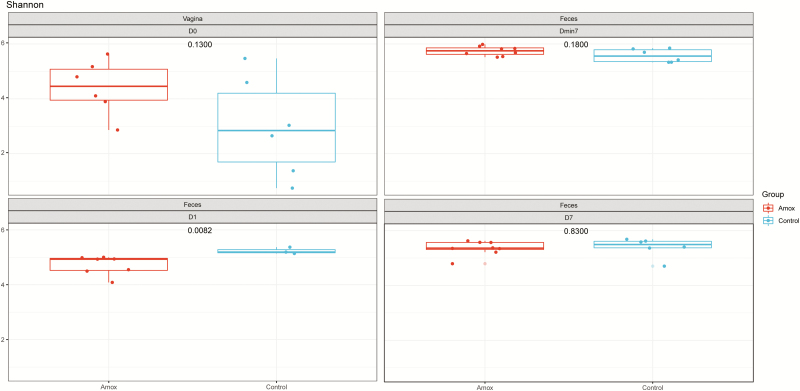
Shannon diversity of vagina and fecal samples of amoxicillin and control sows. Left top panel depicts the sow vagina microbiota diversity for amoxicillin (red) and control (blue). Whereas the right top panel depicts the sow fecal microbiota diversity at day −7, i.e., 7 d before starting the antibiotic treatment. Left bottom panel shows day 1, i.e., 1 d after cessation of the antibiotic treatment, and the right bottom panel shows 7 d after cessation of the antibiotic treatment. The numbers in the center of each panel represent the *P*-value as calculated by a Student’s *T*-test comparing the Amox vs. the control group.

**Figure 4.**
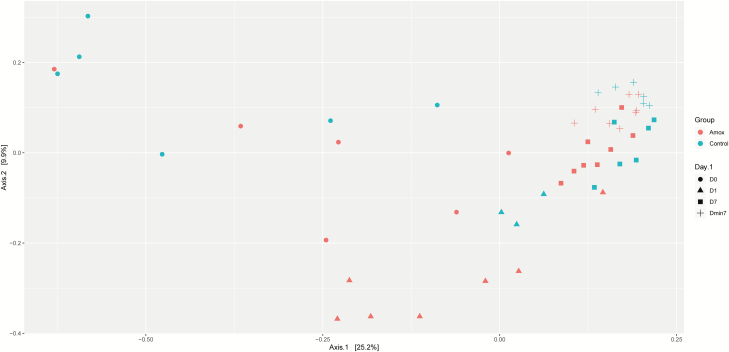
Principal coordinate analysis of the vagina and fecal samples of amoxicillin and control sows. Groups are depicted by different colors, where red represents amoxicillin-treated sows and blue represents control sows. The site of sampling and day are depicted by shape, filled circle, for vagina day 0 (V_D0), + for feces day −7 (7 d before starting the antibiotic treatment, F_Dmin7), filled triangle, for feces day 1 (1 d after cessation of the antibiotic treatment, F_D1), and filled square, for day 7 (7 d after cessation of the antibiotic treatment, F_D7). The first axis explains 25.2% of the variation, whereas the second axis explains 9.9%.

The effect of maternal amoxicillin treatment on microbiota composition and diversity of both sow and piglet was determined. Amoxicillin treated sows showed a numerical reduction in bacterial concentration in their vagina on day 0, as well as in feces on day 1 compared with control sows (Figure 2a), although these differences were not statistically significant (*P* > 0.05 after post hoc testing of the two-way ANOVA). There was a significant impact of antibiotic treatment on the Shannon diversity of the fecal microbiota of sows on day 1, but not on day −7 or day 7 or the vaginal microbiota at partus (Figure 3). Although diversity of sow fecal microbiota decreased after farrowing, this was true for both the antibiotic treated sows and the control group, suggesting that giving birth affected fecal microbiota composition. One week after gestation (and cessation of antibiotic therapy) sow fecal microbial diversity was already back at the original diversity (from day −7). The vaginal microbiota of sows was less diverse than the fecal microbiota (Figure 3). When focusing on the total microbiota composition of the different sites, fecal vs. vaginal, we observed significant differences for the day (days −7, 0, 1, and 7) and group interaction (amoxicillin/control) (Figure 4). This means that we cannot disentangle the effect of day and group.

**Figure 5.**
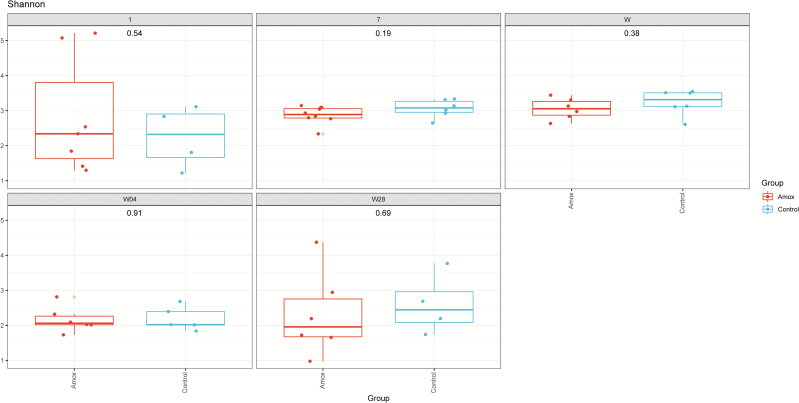
Shannon diversity of jejunal samples of amoxicillin and control piglets. Each panel represents a different day, from top left to bottom right, days 1, 7, weaning, 4 d postweaning, and 28 d postweaning. Red depicts offspring from amoxicillin-treated sows and blue depicts offspring from control sows, the value in the center represents the *P*-values.

**Figure 6.**
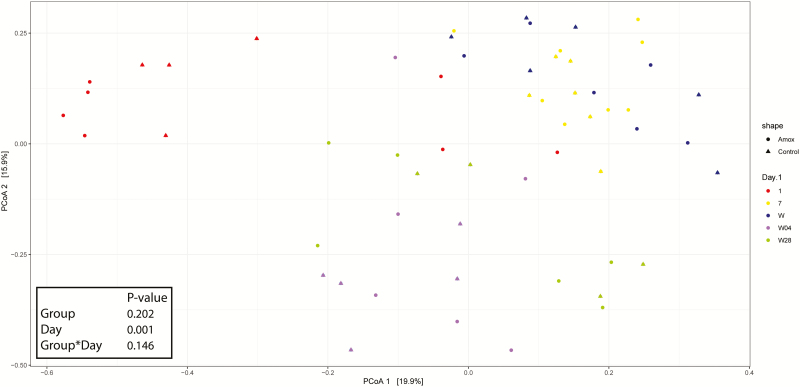
Principal coordinate analysis of jejunal microbiota composition of offspring from amoxicillin-treated sows or control sows. Each dot represents data from one animal. Colors indicate the age the piglets belong to; red, day 1 piglet jejunum; yellow, day 7 piglet jejunum; dark blue, day W piglet jejunum; lilac, day W28 piglet jejunum; and light green, day W4 piglet jejunum. Whereas the shape corresponds to the treatment group, control (triangle) and amoxicillin (circle) piglets. In the bottom left, the *P*-values for Group, Day, and Group × Day are depicted resulting from the “Permutational Multivariate Analysis of Variance Using Distance Matrices.”

In piglets, jejunal ingesta did not show any significant changes in Shannon diversity between control piglets and piglets from sows that received amoxicillin during gestation (Figure 5). There was however a clear time effect observed in the diversity profile. At day 1, immediately after birth, Shannon diversity and “observed species” were low compared with later time points. Until weaning, Shannon diversity and “observed species” increased in time to both drop steeply at weaning (Figures 2b and 5). After weaning, diversity and amount of microbiota increased again but at a slower rate than before weaning. Four days after weaning, piglets from amoxicillin-treated sows showed a significant decrease in the “observed species” compared with control piglets; however, this did not result in a change in the Shannon diversity. The overall composition of jejunal microbiota did not change due to the amoxicillin treatment; however, the day (time point) was significant (Figure 6).

**Figure 7.**
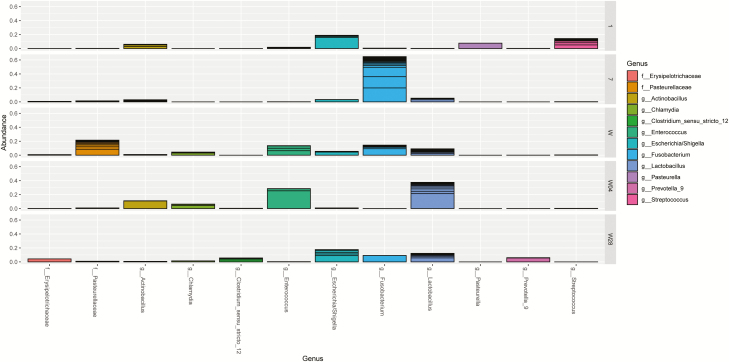
Top 10 bacterial groups at genera level in average abundance across Group and Day in piglets born from amoxicillin or control sows. From left to right on the *x*-axis are *Erysipelotrichaceae*, *Pasteurellaceae*, *Actinobacillus*, *Chlamydia*, Clostridium_sensu_stricto_12, *Enterococcus*, *Escherichia*/*Shigella*, *Fusobacterium*, *Lactobacillus*, *Pasteurella*, Prevotella_9, and *Streptococcus*. The days are depicted from top to bottom, days 1, 7, weaning (W, day 26), 4 d postweaning (W4), 28 d postweaning (W28), respectively, where in each graph the average relative abundance (ranging from 0 to 1) is depicted.

**Figure 8.**
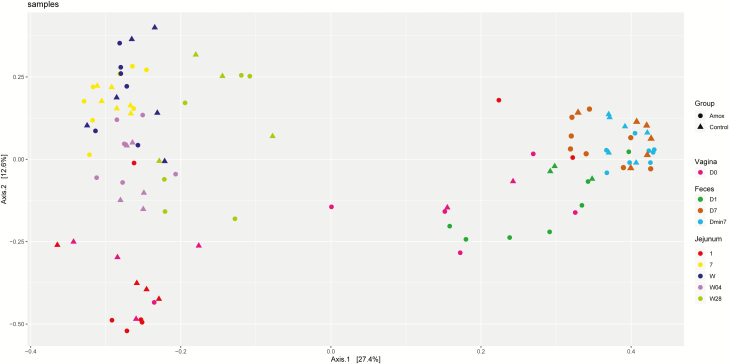
Principal coordinate analysis of all microbiota samples based on Bray–Curtis dissimilarities. Each dot represents one animal. Groups are depicted by different symbols, where circles represent amoxicillin-treated sows and triangles represent control sows. Color depicts the sampling site and day: magenta, day 0 (birth offspring) sow vagina red; dark green, day 1 after cessation amoxicillin treatment sow feces; brown, day 7 after cessation amoxicillin treatment sow feces; light blue, 7 d before start amoxicillin treatment sow feces; red, day 1 piglet jejunum; yellow, day 7 piglet jejunum; dark blue, day W piglet jejunum; lilac, day W28 piglet jejunum; and light green, day W4 piglet jejunum.

This clearly shows the development of the jejunal microbiota. When focusing on the 10 most relatively abundant bacterial genera, the results show the development and changes in time of the jejunal microbiota (Figure 7). At day 1, a high percentage for the *Escherichia*/*Shigella* and *Streptococcus* group compared with all other time points. At day 7, a high percentage of *Fusobacterium* was present, whereas at weaning no bacterial groups were predominantly present. At 4 d after weaning the groups *Lactobacillus* and *Enterococcus* were highest, and at 4 wk after weaning again values were similar as observed at weaning. There was one exception: the *Pasteurellaceae* group was almost absent at 4 wk after weaning. Principle coordinate analysis (**PCoA**) of Bray–Curtis dissimilarities indicated overlap between maternal vaginal microbiota and piglet jejunal microbiota on day 1 (Figure 8). An association between these two microbial populations was observed, suggesting the first colonization of the neonatal jejunum originates from the maternal birth canal.

### Effect of maternal antibiotic treatment on neonatal mucosal gene expression

**Figure 9.**
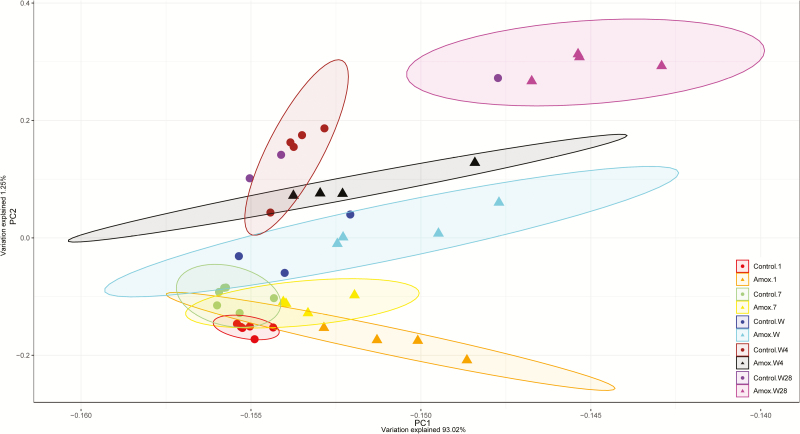
Principal component analysis of jejunal gene expression of offspring from sows that either received amoxicillin treatment or belonged to the control group. The variation explained on PC1 is 93.02% and for PC2 1.25%. Each dot represents data from 1 animal, triangles represent piglets from amoxicillin-treated sows; circles represent piglets from control sows. Colors indicate the age and group the piglets belong to; orange is day 1 amoxicillin (amox), red is day 1 control, yellow is day 7 amox, green is day 7 control, cyan is day weaning (W; d26) amox, blue is W control, black is 4 d postweaning (W4) amox, brown is W4 control, magenta is day 28 postweaning (W28) amox, purple is W28 control. Note that no ellipses could be drawn for control W (d26) and control W28, because only three piglets remained after quality control.

The effect of maternal antibiotic treatment on intestinal jejunal gene expression of offspring was determined at day 1, day 7, weaning (day 26), 4 d postweaning (day 30), and 4 wk postweaning (day 56). Large numbers of genes were affected in expression level (*P*_adj_ < 0.05; log fold change > |1.5|), starting immediately after birth (day 1) where 62 genes were significantly differentially expressed until 4 wk postweaning (day 56) where 223 genes were differentially expressed. The highest number of differentially expressed genes was found at weaning (*n* = 243), whereas no significant differences in gene expression were found on day 7. Principal component analysis showed that besides treatment, time was also a strong parameter determining gene expression patterns for both groups (Figure 9). On all time points, expression patterns from piglets from control sows were separated from piglets from amoxicillin treated sows. At day 7 however, all samples clustered closely together, confirming the lack of differentially expressed genes at that time point. Intestinal development as function of time was suggested by the changing expression patterns in time (Figure 9). At day 54, both groups were strongly separated. To get more insight into the biological processes that were regulated in the jejunal scrapings of piglets, annotated genes were included in further functional pathway-enrichment analyses using DAVID (Table 2). At all time points, except for day 7, after FAC several processes were differentially regulated in piglets from amoxicillin-treated sows compared with piglets from control sows. Although some processes were regulated at different time points, most processes were specific for a certain time point. Regulated processes included metabolic processes, apoptosis, mitotic cell cycling, and mitosis (*P*_adj_ < 0.05; log fold change > |1.0|) (Table 2).

**Table 2:** FAC of regulated genes in jejunal scrapings of piglets from sows from the amoxicillin group compared to the control group

Condition	Day	Number of clusters	Generalized term^1^	Number of genes^2^	Enrichment score^3^
Upregulated genes					
Amoxicillin vs. Control	1	2	Immunoglobulin domain	5	1.14
			Cell junction	4	1.001
Amoxicillin vs. Control	W	2	Transmembrane	56	1.65
			Regulation of cell activation	5	1.258
Amoxicillin vs. Control	W + 4d	0	—	0	—
Amoxicillin vs. Control	W + 4w	1	Appendage morphogenesis	3	1.357
Downregulated genes					
Amoxicillin vs. Control	1	4	Ion channel activity	5	1.64
			Ion transport	4	1.42
			Blood circulation	3	1.23
			Membrane fraction	6	1.2
Amoxicillin vs. Control	W	24	Regulation of protein metabolic process	9	2.65
			Apoptosis	17	2.17
			Negative regulation of macromolecule	20	2.12
			Organelle lumen	30	2.12
			Intracellular protein transport	13	2.12
			Macromolecule catabolic process	20	2.05
			Initiation factor	5	1.77
			GRAM	3	1.74
			Nucleotide-binding	33	1.45
			Protein catabolic process	5	1.45
			Regulation of apoptosis	17	1.41
			Embryonic limb morphogenesis	5	1.38
			Phosphate metabolic process	12	1.28
			Domain:Helicase C-terminal	5	1.25
			RNA catabolic process	4	1.17
			Bromodomain	3	1.17
			Vacuole	8	1.16
			Glycoprotein biosynthetic process	6	1.16
			Cytoplasmic vesicle	14	1.12
			Ubiquitin-protein ligase	5	1.09
			Macromolecular complex assembly	14	1.1
			Regulation of transcription	9	1.08
			Sequence motif:DEAD box	3	1.06
			GTPase activity	7	1.05
Amoxicillin vs. Control	W + 4d	4	Ribosome	5	2.63
			Cell cycle process	7	1.34
			Ubiquitin-dependent protein	3	1.09
			M phase	5	1.09
Amoxicillin vs. Control	W + 4w	14	Purine nucleoside binding	46	4.69
			Phosphorylation	25	2.73
			Serine/threonine-protein kinase	12	2.53
			Mitotic cell cycle	15	2.26
			Mitosis	9	1.67
			Repeat:RCC1 5	3	1.59
			Macromolecule catabolic process	20	1.47
			Zinc finger	3	1.46
			Cell fraction	21	1.42
			Endoplasmic reticulum part	10	1.4
			Helicase activity	6	1.27
			Organelle lumen	31	1.24
			Transcription	8	1.22
			Nucleotide kinase activity	3	1.13

^1^classification stringency was set at high; ^2^ adjusted p value < 0.05 and log Fold Change > |1.5|; ^3^ clusters were indicated significant if above 1.00

### Maternal antibiotic treatment has developmental effects on gut morphology in offspring

**Figure 10.**
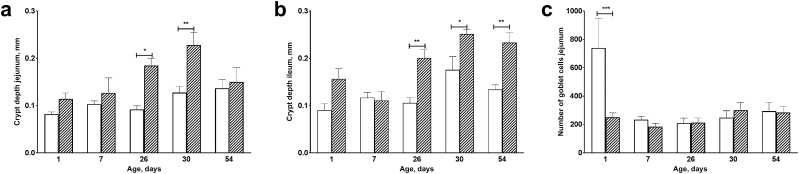
Intestinal morphometry of piglets from sows treated with amoxicillin or control sows. Crypt depth in jejunum (panel a) and ileum (panel b) as well as goblet cells in jejunum tissue (panel c) were determined in piglets from sows treated with amoxicillin (hatched bars) or from control sows (open white bars). Error bars indicate SEM. * *P* < 0.05; ** *P* < 0.01; *** *P* < 0.001.

There were no significant differences in villus length nor in mucosa length, indicating that the absorptive capacity was comparable between treatments. There was a significant difference in crypt depth between the treatment groups, where piglets from amoxicillin treated sows showed deeper crypts (Figure 10a). In the ileal mucosa also significantly deeper crypts were found (Figure 10b).

To determine whether the observed differences in development are also reflected by differences in proliferation, PCNA staining was used as a proliferation marker. However, no differences in proliferation of intestinal cells were found between the treatment groups. Numbers of goblet cells present in the jejunal mucosa significantly differed on day 1 (Figure 10c). Piglets that were born from control sows had much larger numbers of jejunal goblet cells. This difference in goblet cells was not found in the ileum.

## Discussion

Our study shows that maternal antibiotic treatment during the last week of gestation affects intestinal development in offspring for a period of at least 7 wk. Significant effects of the maternal antibiotic treatment on the microbial colonization and development of the gut in the offspring animals were observed although the maternal antibiotic treatment did not affect any of the measured performance parameters of the piglets. The strongest effects on intestinal development, as measured by gene expression profiles, were identified close to birth (day 1) and around weaning (day 26). Since those are important transition moments, discussion of the results was focused on these time points.

### Transition moment: birth

Different bacterial populations were changed in the vaginal microbiota at birth (day 0) and this population was comparable with (sow) fecal microbiota at day 1. On the first day of life, jejunal microbiota is very diverse, probably reflecting the early colonizers of the naïve intestine. Although little is known about transmission routes of colonizing organisms, it is very likely that piglets are colonized from maternal microbiota either via the vagina or ingestion of sow feces or colostrum (Blaser and Dominguez-Bello, 2016). Therefore, maternal antibiotic treatment could impact the early life colonization of the neonate by influencing the first colonizers (Schulfer and Blaser, 2015). In addition to the microbial changes, the piglets from antibiotic-treated sows showed increased expression of genes involved in the processes “tight junctions” and “immunoglobulins” immediately after birth. The tight junction proteins, including claudin3 and occludin, contribute to the barrier function of the intestine (Caricilli et al., 2014). At birth the gut of neonates is still “open” to allow passive uptake of macromolecules such as maternal antibodies from colostrum and milk in the first days of life (Pasternak et al., 2015). The formation of tight junction proteins closes the gut to form a solid barrier that protects the host from external challenges such as pathogens. The observed upregulation of these tight junctions may imply that the gut of neonates from amoxicillin treated sows closes earlier in life, thus putatively preventing uptake of macromolecules. Tight junction protein expression, i.e., occludin, was previously shown to be affected by nutritional interventions, where formula fed piglets displayed increased tight junction expression compared with sow fed piglets (Huygelen et al., 2012), indicating that oral interventions can affect expression of tight junctions proteins. At the same time point immediately after birth, it was shown that the number of jejunal goblet cells was significantly reduced in piglets from antibiotic treated sows. Goblet cells produce mucus that forms a thick protective layer covering the epithelial cells. This layer forms a dynamic protective barrier against invading pathogens, and simultaneously creates a niche where more beneficial bacteria can survive (Kim and Khan, 2013). Piglets from the antibiotic treated sows have significant lower numbers of jejunal goblet cells at birth, which could result in the observed slightly different microbiota on day 1. Although the production of mucus was not determined in this study, the significant decrease in jejunal goblet cells may decrease the protective capacity of the mucus layer against pathogens early in life. This thickness of the mucus layer could affect the timing of the regulation of the tight junction, with a mucus layer as first defense, the tight junction function could be delayed, due to less interaction with the lumen of the intestine. Genes associated with “Immunoglobulins” were upregulated in the piglets from amoxicillin treated sows. Immunoglobulins are involved in recognizing antigens, facilitating processes like opsonization and phagocytosis. The specific humoral immunity of neonatal animals mainly comes from maternal antibodies in milk. With ageing and organ development, the animal’s own immune system maturates and induces synthesis of its own specific antibodies (Butler et al., 2017). The increased expression of immunoglobulin-related genes after birth, most likely reflects the postnatal development of B-cells, a prerequisite for immunoglobulin production, since at this stage, piglets are still unable to produce specific antibodies although natural antibodies are present at that time. It could be speculated that the upregulation of the biological process of “immunoglobulins” is linked to the putative diminished passive uptake due to the earlier closure of the gut. However, additional research focused on immunoglobulin concentrations and tight junction formation in neonatal piglets as function of time is required to demonstrate this.

In summary, upregulation of genes associated with “tight junctions” and “immunoglobulins” immediately after birth will probably lead to intestinal epithelium that is more rapidly closed in piglets from amoxicillin-treated sows, whereas they have lesser numbers of goblet cells able to produce a mucin barrier. Thus antibiotic treatment may negatively affect the development of the first line of defense in neonates from amoxicillin-treated sows. A recent publication has demonstrated that transient maternal colonization during gestation also has strong impact on the intestinal development of the immune system of the offspring (Gomez de Aguero et al., 2016). This may explain our results showing that not only maternal microbiota composition was affected but also barrier function and intestinal immunological programming of neonates.

### Transition moment: weaning

At the second transition moment, i.e., weaning, it is known that the microbiota adapts to the transfer from liquid milk-based diets to solid feeds with vegetable protein and energy sources (Su et al., 2008). Intestinal microbiota benefit from the nutrient-rich environment of the gut, whereas the host benefits from microbiota-mediated adaptive digestive efficiency (Hooper et al., 2012; Maynard et al., 2012). At the transition moment at weaning, microbial diversity decreases due to the change of diet, combined with the abrupt cessation of exposure to sow milk, as well as stress associated to being removed from the sow. After weaning the permanent microbiota starts to develop which is reflected in an increasing diversity, this secondary succession was irrespective of treatment. This time-dependent pattern of diversity was also described by others (Unno et al., 2014). Our data corroborate these findings, piglets at weaning and following weeks showed profound changes in gene expression in the gut wall. Gene expression analysis showed that networks of genes that were differentially expressed between intestinal scrapings of piglets from amoxicillin-treated sows and control sows are involved in growth and development of cells, such as apoptosis, metabolic changes, and mitosis. These changes suggest that intestinal development is differentially regulated between the treated groups. These suggestions are confirmed by the observed morphological changes in the intestine, showing that around weaning piglets from amoxicillin-treated sows have significantly deeper crypts in both jejunum and ileum, but no difference in villus height or cells replication. Taken together, gene expression and morphological data indicate that the crypts of piglets deepen around weaning as an effect of the amoxicillin treatment which in combination with the upregulation of cell cycle processes, ribosomal activity, and protein degradation imply that the intestinal development, the subsequent differentiation of cells or the timing of these processes was delayed by maternal antibiotic treatment. Since on day 1 immediately after birth differences in barrier function and intestinal integrity were found, it cannot be excluded that these differences steered the intestinal development in another direction, resulting in the described developmental differences observed around weaning. Another hypotheses could be that altered crypt depth might be mediated by microbiota composition in early life, as shown for conventional pigs which display deeper crypts than germ-free or mono-colonized pigs, and in rats a conventional microbiota leads to deeper crypts than in germ-free conditions (Shirkey et al., 2006; Cherbuy et al., 2010; Arnal et al., 2014). These observed changes in intestinal microbiota of offspring are in accordance with the findings of Holman and Chénier, who concluded that there is considerable resilience to antibiotic (administered to the piglets) perturbation of the gut microbiota in pigs over a period of 19 wk from suckling onward (Holman and Chenier, 2014).

## Conclusion(s)

Maternal antibiotic use during (late) gestation, as proof of concept for a modulation, changes the diversity in sow fecal microbiota and also affects the intestinal development of piglets in early life, however, without significant change to the piglets microbiota. This implies that it is possible to modulate the intestinal development of piglets by maternal interventions, although the impact on health of this modulation is not known. Therefore, it may be possible to steer the intestinal development of offspring using maternal (feed) interventions.

## Supplementary Material

skaa181_suppl_Supplementary_Table_S1

## Abbreviations

DAVID: Database for Annotation, Visualization and Integrated Discovery
DNA: deoxyribonucleic acid
FAC: functional annotation clustering
GFP: green fluorescent protein
LIMMA: Linear Models for Microarray and RNA-Seq Data
PCNA: proliferating cell nuclear antigen
PCoA: principle coordinate analysis
PCR: polymerase chain reaction
RNA: ribonucleic acid
