# Supplementary material for: The effect of maternal antibiotic use in sows on intestinal development in offspring
Source: J Anim Sci. 2020 Jun 1;98(6):skaa181. doi: 10.1093/jas/skaa181 (PMC7295330; doi:10.1093/jas/skaa181)
Supplement: skaa181_suppl_Supplementary_Table_S1 [file skaa181_suppl_supplementary_table_s1.docx]

**Supplementary Table S1** Composition of gestation and lactation feed

| *Ingredient* | *Unit* | *Gestation Diet* | *Lactation Diet* |
| --- | --- | --- | --- |
| Wheat |  | 10.00 | 10.00 |
| Betain 96% |  | 0.10 | 0.20 |
| PHYZYME XP 5000 TPT |  | 0.01 | 0.01 |
| Sow premix |  | 1.00 | 1.00 |
| Maize |  | 26.48 | 29.93 |
| Barley |  | 20.00 | 20.00 |
| Soy bean meal 49, Cf |  | 2.45 | 11.54 |
| Sunflower seed meal |  | 12.21 | 14.64 |
| Beet pulp, Sug <10% |  | 5.00 | 0.00 |
| Soya hulls, CFiber 340 |  | 20.00 | 5.85 |
| Limestone |  | 0.48 | 1.32 |
| Monocalcium phosphate |  | 0.37 | 0.68 |
| Na bicarbonate |  | 0.33 | 0.43 |
| Soya oil |  | 1.24 | 3.86 |
| L-Lysine HCl 98% |  | 0.06 | 0.25 |
| L-Threonine 98% |  | 0.00 | 0.03 |
| Choline Chloride 50% |  | 0.08 | 0.08 |
| Biotine-Mix |  | 0.15 | 0.15 |
| Vitamin E 50% adsorb |  | 0.04 | 0.04 |
| Calculated values |  |  |  |
| DM | g/kg | 883 | 887 |
| CP | g | 140 | 180 |
| Ash | g | 53 | 61 |
| CF | g | 113 | 65 |
| EE | g | 38 | 63 |
| NDF | g | 238 | 159 |
| Starch am | g | 329 | 350 |
| ADL | g | 13 | 12 |
| C18:0 | g | 1.0 | 1.9 |
| C18:1 | g | 7.5 | 12.8 |
| C18:2 | g | 18.0 | 30.9 |
| C18:3 | g | 1.7 | 3.5 |
| dEB | meq | 210 | 220 |
|  |  |  |  |
| Net Energy | Kcal/kg | 2037 | 2310 |
|  |  |  |  |
| LYS | g | 6.2 | 9.6 |
| MET | g | 2.5 | 3.1 |
| MET+CYS | g | 5.1 | 6.3 |
| THR | g | 5.0 | 6.7 |
| TRP | g | 1.5 | 2.0 |
| SID ILEpig | g | 4.4 | 6.1 |
| SID LEUpig | g | 8.5 | 11.6 |
| SID LYSpig | g | 4.8 | 8.4 |
| SID M+Cpigs | g | 4.2 | 5.4 |
| SID METpig | g | 2.2 | 2.8 |
| SID THRpig | g | 3.9 | 5.6 |
| SID TRPpig | g | 1.2 | 1.7 |
| SID VALpig | g | 5.3 | 7.2 |
| Ca | g | 6.5 | 9.5 |
| Ca / P |  | 1.5 | 1.6 |
| P | g | 4.5 | 5.9 |
| IP | g | 0.5 | 1.0 |
| dP swine | g | 2.4 | 3.3 |
| I | mg | 1.19 | 1.11 |
| Cl | g | 2.9 | 3.3 |
| K | g | 7.5 | 7.9 |
| Na | g | 2.3 | 2.5 |
| Mg | g | 2.2 | 2.3 |
| Cu | mg | 23 | 23 |
| Mn | mg | 50 | 49 |
| Fe | mg | 390 | 342 |
| Se | mg | 0.30 | 0.30 |
| Zn | mg | 136 | 135 |
| Vitamin A | I.U. | 10000 | 10000 |
| Vitamin D | I.U. | 2,000 | 2,000 |
| Vitamin E | I.U. | 60 | 60 |
| Vitamin K | mg | 2 | 2 |
